# Retinal thickness as a marker of disease progression in longitudinal observation of patients with Wolfram syndrome

**DOI:** 10.1007/s00592-017-1042-6

**Published:** 2017-08-30

**Authors:** Agnieszka Zmyslowska, Wojciech Fendler, Arleta Waszczykowska, Anna Niwald, Maciej Borowiec, Piotr Jurowski, Wojciech Mlynarski

**Affiliations:** 1 Department of Pediatrics, Oncology, Hematology and Diabetology, Medical University of Lodz, Sporna Str. 36/50, 91-738 Lodz, Poland; 2 Department of Biostatistics and Translational Medicine, Medical University of Lodz, Lodz, Poland; 3 Department of Radiation Oncology, Dana-Farber Cancer Institute, Boston, MA USA; 4 Department of Ophthalmology and Vision Rehabilitation, Medical University of Lodz, Lodz, Poland; 5 Outpatient Clinic of Ophthalmology, Central Clinical Hospital, Lodz, Poland; 6 Department of Clinical Genetics, Medical University of Lodz, Lodz, Poland

**Keywords:** Monogenic diabetes, Wolfram syndrome, Ophthalmology complications, Retina

## Abstract

**Aims:**

Wolfram syndrome (WFS) is a recessively inherited monogenic form of diabetes coexisting with optic atrophy and neurodegenerative disorders with no currently recognized markers of disease progression. The aim of the study was to evaluate retinal parameters by using optical coherence tomography (OCT) in WFS patients after 2 years of follow-up and analysis of the parameters in relation to visual acuity.

**Methods:**

OCT parameters and visual acuity were measured in 12 WFS patients and 31 individuals with type 1 diabetes.

**Results:**

Total thickness of the retinal nerve fiber layer (RNFL), average retinal thickness and total retinal volume decreased in comparison with previous OCT examination. Significant decreases were noted for RNFL (average difference −17.92 µm 95% CI −30.74 to −0.10; *p* = 0.0157), macular average thickness (average difference −5.38 µm 95% CI −10.63 to −2.36; *p* = 0.0067) and total retinal volume (average difference −0.15 mm^3^ 95% CI −0.30 to −0.07; *p* = 0.0070). Central thickness remained unchanged (average difference 1.5 µm 95% CI −7.61 to 10.61; *p* = 0.71). Visual acuity of WFS patients showed a strong negative correlation with diabetes duration (*R* = −0.82; *p* = 0.0010). After division of WFS patients into two groups (with low-vision and blind patients), all OCT parameters except for the RNFL value were lower in blind WFS patients.

**Conclusions:**

OCT measures structural parameters and can precede visual acuity loss. The OCT study in WFS patients should be performed longitudinally, and serial retinal examinations may be helpful as a potential end point for future clinical trials.

## Introduction

Wolfram syndrome (WFS) (OMIM 222300) is caused by recessive mutations mainly in the *WFS1* gene on chromosome 4p16.1 [1, 2]. Wolframin, the protein product of *WFS1*, is an important part of the endoplasmic reticulum (ER) membrane and its loss of function in neuroendocrine cells increases ER stress, leading to apoptosis and neurodegeneration [3]. At present there is no causal treatment for WFS, and the disease leads to premature death of the patients [4].

The first clinical signs of WFS are diabetes mellitus (DM) and optic atrophy (OA). Typically, the onset of insulin-dependent DM precedes the diagnosis of OA, by a margin of 4 years on average [5–7]. Other features of the syndrome, which develop as the patient gets older, are: progressive blindness and deafness, diabetes insipidus, urodynamic and endocrinological disorders, and psychiatric and neurological abnormalities [8]. In addition to OA, other vision abnormalities (maculopathy, retinopathy and cataract) may emerge, contributing to impairment and loss of vision [9, 10]. Moreover, very recent studies on Wfs1-deficient mice disclosed significant dysfunction of the visual system, associated with ER stress in the retina [11]. These facts support the need to perform studies focused on neurodegenerative changes in the retina among patients with WFS. The best way to evaluate retinal integrity is by using optical coherence tomography (OCT), which can non-invasively detect pathology of the optic nerves and also images the posterior pole of the eye [12]. Therefore, we focused on the clinical features of WFS and combined them with the OCT results suggesting that a retinal thinning could serve as a biomarker of WFS disease progression [13]. Over time, visual acuity of WFS patients becomes worse which can exclude accurate estimation of some OCT parameters [14]. However, a long-term retina evaluation seems to be clinically useful. Thus, the aim of the study was to evaluate retinal parameters by using OCT after 2 years of follow-up and analysis of the parameters in relation to visual acuity in patients with WFS.

## Materials and methods

Before initiating the study, the Bioethics Committee of the Medical University of Lodz approved the study protocol and all patients or parents of underage participants expressed written consent for participating in the study. The present study included two groups: 12 patients with WFS and a control group composed of 31 individuals with type 1 diabetes (T1D). Among the 12 patients with WFS, 10 were a prospectively followed-up group whose initial data were published previously [13]. The remaining two individuals at follow-up analysis were newly recruited WFS patients without prior OCT examinations. The average duration of follow-up in the WFS group was 22 ± 2.2 months. The T1D group was recruited as controls for the WFS individuals among children treated in the study center from children consecutively hospitalized within the Department of Pediatrics, Oncology, Hematology and Diabetology between October 2015 and January 2016. Only children with duration of diabetes longer than 3 years admitted for routine metabolic control assessment were recruited to the study.

Diabetes was recognized typically, according to WHO criteria. At onset of T1D, autoantibodies and decreased C-peptide level were detected in all patients. HbA1c was determined by high-performance liquid chromatography (HPLC) using the Bio-Rad VARIANT™ Hemoglobin A1c Program (Bio-Rad Laboratories, Inc. Hercules, CA, USA) with its values represented as percentages and mmol/mol.

All WFS patients had DM recognized according to WHO criteria, OA recognized using visual evoked potentials (VEP) and/or MRI, and confirmed during the first OCT study. Detailed characteristics of the study groups are shown in Table 1. The causative mutations in the *WFS1* gene detected in all WFS patients were recognized as described previously [14].

**Table 1:** General characteristics of the studied groups

Group	Sex M/F	Age at OCT study and visual acuity examinations average ± SD (years)	Age at diabetes diagnosis average ± SD (years)	Duration of diabetes average ± SD (years)	HbA1c average ± SD (%/NGSP/mmol/mol IFCC)
WFS *N* = 12	3/9	18.58 ± 4.48	6.19 ± 2.99	12.38 ± 4.86	7.86 ± 1.12/62 ± 12.5
T1D *N* = 31	13/18	12.63 ± 3.36	6.70 ± 3.58	5.82 ± 2.88	7.29 ± 0.62/56 ± 7

*WFS* Wolfram syndrome, *T1D* type 1 diabetes, *SD* standard deviation

### OCT study

All first OCT examinations in WFS patients were conducted in the Clinic of Ophthalmology of the Central University Hospital in Lodz, Poland, with the Topcon 3D Optical Coherence Tomograph 2000 (Fastmap version 8.11.003.04, Japan). The second OCT study was performed in the patients from the study and control groups in the Department of Ophthalmology and Vision Rehabilitation of the Medical University of Lodz, Poland, with Topcon 3D Optical Coherence Tomograph 1000 (MARK II, version 3.51, Topcon Inc., Paramus, NJ, USA). All studies were carried out with subjects in a resting position after an average of 15–20 min after inducing mydriasis and evaluated independently by two experienced ophthalmologists. We determined the following parameters: total thickness of the retinal nerve fiber layer (RNFL), average retinal thickness, central thickness and total retinal volume using 3D disk and 3D macula scans with a 6 × 6 mm area. Owing to absence of visual fixation in patients with OA, three parameters were measured in all WFS patients: average retinal thickness, central thickness and total volume. Averages from measurements in both eyes were calculated and used for further analysis.

### Visual acuity

All the patients underwent at follow-up an assessment of best spectacle-corrected visual acuity (BSCVA) for distant vision measured using Snellen charts at a decimal scale (LCD Frey CP-400, Frey Sp.J., Piaseczno, Poland). The examination was conducted independently by two experienced ophthalmologists. All T1D patients fulfilled the criteria for correct vision (>0.3). Depending on the results of visual acuity, WFS patients were divided into patients with low-vision (0.05–0.3) and blind patients (<0.05).

### Statistical analysis

Paired comparisons were performed with a Wilcoxon’s signed rank test for matched samples. Correlations were evaluated with a Spearman’s rank correlation test. Generalized linear regression model was used to adjust for confounding factors according to the standard methodology of accounting for the impact of such variables as described in [15]. STATISTICA 13.0 software (Statsoft, Tulsa, OK, USA) was used for analysis. A type 1 error probability of 0.05 was chosen as the threshold of statistical significance.

## Results

**Fig. 1:**
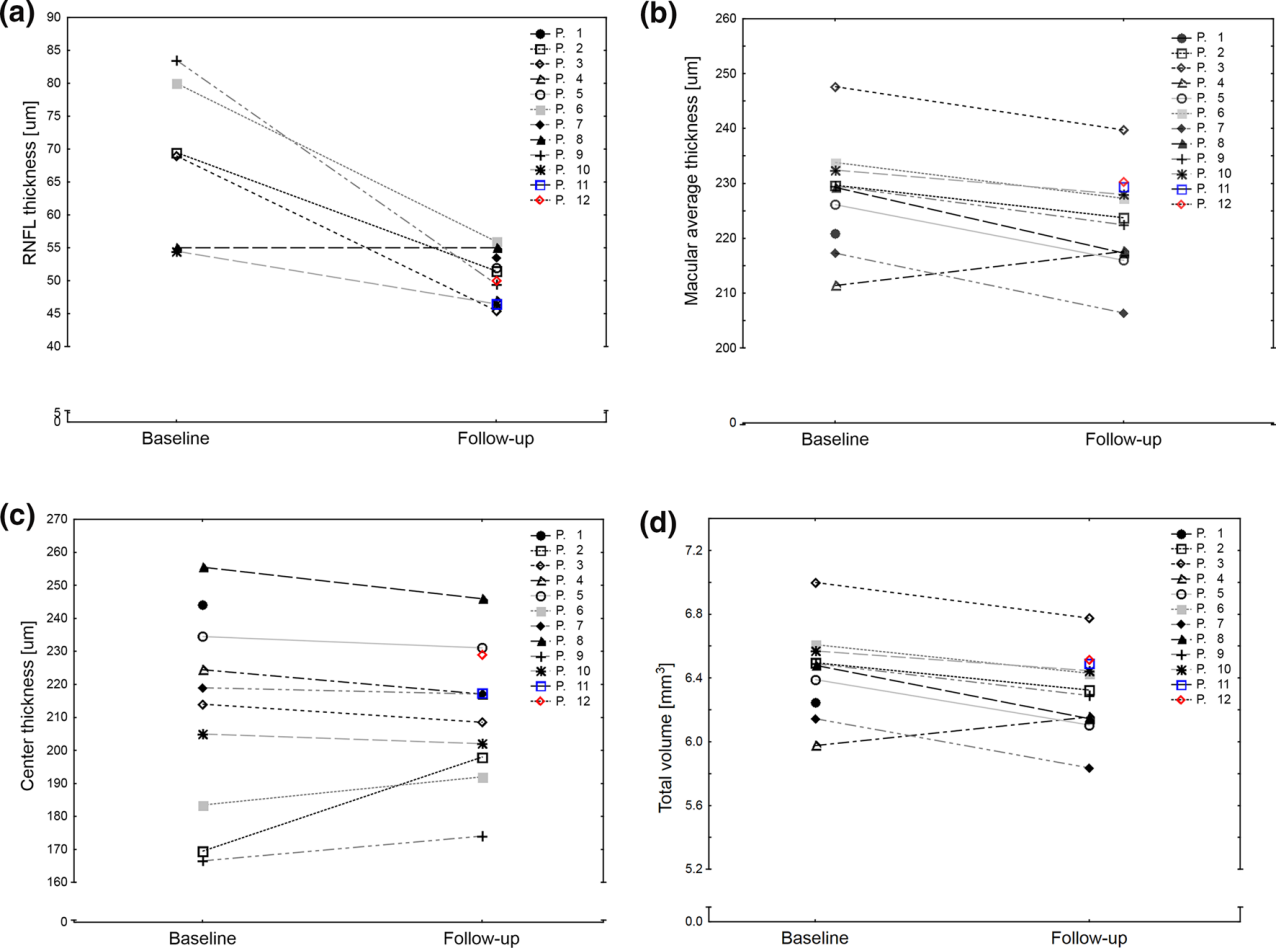
OCT parameters observed in patients with Wolfram syndrome at the first examination and 2-year follow-up observation. Significant decreases were noted for RNFL (average difference −17.92 µm 95% CI −30.74 to −0.10; *p* = 0.0157 (**a**), macular average thickness (average difference −5.38 µm 95% CI −10.63 to −2.36; *p* = 0.0067 (**b**) and total retinal volume (average difference −0.15 mm^3^ 95% CI −0.30 to −0.07; *p* = 0.0070 (**c**). Central thickness remained unchanged (average difference 1.5 µm 95% CI −7.61 to 10.61; *p* = 0.71 (**d**)

**Fig. 2:**
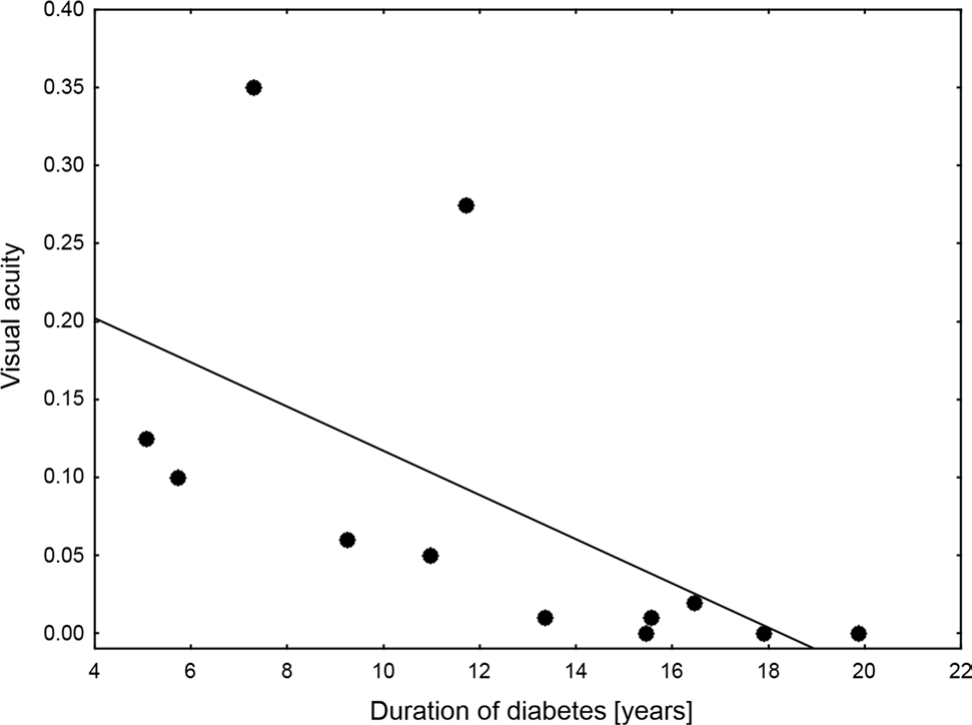
Correlations between visual acuity measured at follow-up and duration of diabetes in Wolfram syndrome patients

Analysis of OCT data showed that in patients with WFS, significant thinning of the RNFL, average retinal thickness and total retinal volume decreased in comparison with the previous examination (Fig. 1a–c). Central thickness remained unchanged (Fig. 1d). Average decrease of RNLF equaled 12.90%/year 95% CI 4.22–21.52%, of average macular thickness 1.54%/year 95% CI 0.535–2.55% and 1.53%/year 95% CI 0.51–2.55% of total retinal volume. All four studied OCT parameters of patients with WFS were consistently lower than ones observed in patients with T1D (Table 2) and they—except for the RNFL value—correlated with OCT parameters from the first examination 2 years ago (Table 3). Then, we evaluated visual acuity of patients with WFS at follow-up examination. Median value equaled 0.083 with three individuals showing complete lack of vision apart from a sense of light in both eyes. Visual acuity of patients with WFS showed a strong negative correlation with the duration of diabetes (Spearman’s *R* = −0.82; *p* = 0.0010, Fig. 2) and correlated with patients’ age, but the difference did not reach statistical significance (*R* = −0.44; *p* = 0.1558). Finally, we divided WFS patients to the patients with low-vision and blind patients. Among OCT parameters, only RNFL remained similar in the subgroups, whereas other retinal parameters differed significantly (Table 4).

**Table 2:** Comparison of OCT data between patients with WFS and T1D at follow-up study

	WFS		T1D		*p* level*
	Average	SD	Average	SD	
RNFL	50.27	3.64	94.59	13.74	0.000001
Macular average thickness (µm)	223.48	8.96	285.31	13.72	0.000001
Center thickness (µm)	212.00	20.10	259.00	20.00	0.000005
Total macular volume (mm^3^)	6.32	0.25	10.27	0.49	0.000001

*WFS* Wolfram syndrome, *T1D* type 1 diabetes, *SD* standard deviation, *RNFL* retinal nerve fiber layer

* Adjusted for age and duration of diabetes

**Table 3:** Correlations between OCT parameters at the first examination and follow-up study in the patients with Wolfram syndrome

	*R*	*p* level
RNFL	0.3143	0.5441
Macular average thickness (µm)	0.8833	0.0016
Center thickness (µm)	0.9791	<0.0001
Total macular volume (mm^3^)	0.8833	0.0016

*RNFL* retinal nerve fiber layer

**Table 4:** Comparison of OCT data between Wolfram syndrome patients with low-vision and blind patients

Parameter	Average	SD	Average	SD	*p* level
	Patients with low vision	Patients with low vision	Blind patients	Blind patients	
RNFL	49.25	3.99687	51.5	3.1225	0.333284
Macular average thickness (µm)	228.4417	6.13526	217.52	8.50512	0.035218
Center thickness (µm)	198.6667	14.93876	228	12	0.006382
Total macular volume (mm^3^)	6.4592	0.17226	6.151	0.24214	0.035724

*RNFL* retinal nerve fiber layer, *SD* standard deviation

## Discussion

For the first time, our study shows that average retinal thickness and total retinal volume of OCT parameters in WFS patients decrease slower than RNFL value. Moreover, since the retinal parameters in WFS patients differed significantly from those observed in patients with T1D after adjustment for the age and duration of diabetes, it is very likely that the changes noted in OCT examinations of WFS patients are the direct consequence of WFS progression rather than of diabetes. It seems, however, that there is a limit value of OCT parameters in WFS patients below of which no retinal thinning progression is observed. Thus, the results show the greatest usefulness of OCT measurements on the early stage of WFS as potential biomarkers of WFS progression. Similar observations were performed by Hoekel et al. evaluating the optical phenotype at a relatively early stage of WFS and making an attempt of correlation with the severity of the disease. Decrease in RNFL during OCT examination correlated in the study with the severity of physical symptoms. However, a lack of correlation between age and reduction in visual acuity was noted making the RNFL value reliable but only the initial marker of WFS progression [16]. Interestingly, our patients at follow-up analysis were an average of 4 years older than patients presented by Hoekel et al. Importantly, in four WFS patients described by Grenier et al. [17] who were about 4.5 years older than our patients, the RNFL thickness was only slightly lower in comparison with our patients.

This is worth noting that a visual fixation by the patient is necessary to measure RNFL during OCT. Thus, the approach cannot be used in some elderly, partially sighted or blind patients. In the present study, by the division of WFS patients depending on the vision acuity we confirmed that RNFL did not differ between groups with low vision and blind, whereas the average retinal thickness and retinal total volume were significantly lower and center thickness was greater in the blind patients. Thus, the best option for the advanced stage of the WFS is to evaluate more stable retinal OCT parameters which could be measured independently of sight fixation.

During follow-up of OCT examination in WFS patients, the significant declines were noted in RNFL, total retinal volume and average thickness, whereas central retinal thickness did not change within 2 years of observation. We can speculate that this finding may be related to the increased risk of maculopathy in these patients or to cellular edema resulting from increased cellular volume due to ER stress.

This also suggested that a loss of retinal neurons is initially reflected by the peripheral thinning of the retinal lining of the eye, while the central thickness remained, at least initially, unaffected. These results are in line with available data about the reduced thickness of the RNFL assessed in OCT studies of neurodegenerative changes occurring in the brain of patients with multiple sclerosis and sleep apnea [18, 19]. Macular thickness is already used as a marker in monitoring treatment of patients with Parkinson’s disease [20], Alzheimer’s disease [21] and age-related macular degeneration (AMD) [22].

Our study also has some limitations. Most importantly, we were not able to recruit a suitably large group of patients with WFS. This is because there are very few such individuals in the Polish cohort recruited through the EURO-WABB rare diseases project [14, 23]. In addition, the first and second OCT studies were conducted on two different devices. However, both were still performed on Topcon 3D Optical Coherence Tomograph, while literature data indicate an objective signal quality of retinal OCT images regardless of the device [24]. Moreover, due to a lack of visual fixation in blind and with very low-vision WFS patients, RNFL thickness was evaluated only in some patients.

Looking at our results, OCT measures structural parameters and can precede visual acuity loss. It should be emphasized that the OCT study in WFS patients should be performed longitudinally, and serial retinal examinations may be helpful as a potential end point for future clinical trials.
